# 24-hour Intraocular pressure monitoring: the way ahead

**Published:** 2019

**Authors:** Shibal Bhartiya, Meenakshi Gangwani, R. Balwant Kalra, Anand Aggarwal, Meghal Gagrani, Kumar Namagiri Sirish

**Affiliations:** * Fortis Memorial Research Institute (FMRI), Gurugram, Haryana, India; ** Chacha Nehru Bal Chikitsalya (CNBC), New Delhi, India; *** Shanti Devi Eye Hospital, Yamunanagar, Haryana, India; **** Department of Ophthalmology, Government Medical College, Amritsar, Punjab India; ***** Dr R.P. Centre for Ophthalmic Sciences, New Delhi, India; ****** Meenakshi Mission Hospital and Research Centre, Madurai, India; N Eye Care, Madurai, India

**Keywords:** IOP diurnal variation, contact lens sensor, rebound tonometry

## Abstract

**Aim:** Large diurnal intraocular pressure (IOP) fluctuation is a single most independent risk factor for glaucoma progression besides raised IOP. The major limitation of Goldman applanation tonometer (GAT) is its inability to measure night IOP without disturbing the patient’s sleep. We discussed the methods available for the 24-hour IOP monitoring and its relevance in glaucoma.

**Methods:** A PUBMED search was performed using the 24 Hour tonometry, newer tonometry devices, contact lens sensors, as keywords and all relevant articles were studied.

**Results and Conclusion:** A number of methods are available for the 24 hour IOP monitoring. These devices allow home monitoring of IOP without affecting the daily routine. These devices, like Rebound tonometry, Contact lens sensor (CLS), etc., were briefly discussed. Triggerfish is one CLS device that has the capability to measure IOP without affecting the patient’s sleep. Besides being safe and easily tolerable, it has shown reproducible results with other tonometry methods. Triggerfish has also been proven the device of choice in measuring IOP in different glaucoma subtypes and determining the efficacy of treatment in them, the only challenge being that it presently provides data on relative IOP rather than absolute IOP. With future research, triggerfish CLS can become an important device to measure the 24 hour IOP values especially in patients whose office measured IOPs seemingly fit in patient’s target range but still the patients’ disease shows glaucomatous progression. The utility of this device in relation to progressive vision loss is a matter of future study.

**Abbreviations:** CCT = Central Corneal Thickness; CLS = Contact lens sensor; GAT = Goldmann Applanation Tonometer; IOP = Intraocular Pressure; NTG = Normal Tension Glaucoma; PACG = Primary angle closure glaucoma; POAG = Primary open angle glaucoma; VAS = Visual Analogue Score

## Introduction

Glaucoma is an optic neuropathy characterized by typical visual field defects and optic nerve head changes, for which intraocular pressure (IOP) is currently the most commonly identified risk factor. Glaucoma is the second leading cause of irreversible blindness worldwide. More than 60 million people in the world were affected by primary glaucoma in the year 2000 [**1**,**2**]. IOP reduction is the only practical approach to control any type of glaucoma, namely open angle, angle closure glaucoma and even secondary glaucomas. It has been estimated that primary angle closure glaucoma (PACG) accounts for nearly 3.2 million cases worldwide with a high prevalence in Asia and is responsible for nearly half of the blindness from glaucoma. Chronic PACG is responsible for early blindness and generalized visual field defects. Fluctuation in circadian IOP is found to be one of the major risk factor for visual field progression in both PACG and primary open angle glaucoma (POAG) patients [**3**,**4**].

## Short term IOP Fluctuations: Implications and measurement

Intraocular pressure (IOP) is an essential modifiable risk factor for glaucoma management. IOP varies throughout diurnal and nocturnal periods. Short-term variation in IOP represents the nyctohemeral pattern and has been related to continuous progression of glaucoma [**4**]. IOP spikes have also been related to progressive visual field loss [**5**]. In patients with an apparently normal IOP, glaucoma progression is associated with wide diurnal variation in IOP. The nocturnal increase in IOP is related to increased episcleral venous pressure presumably because of fluid redistribution, due to the gain of recumbent position during the supine sleeping period [**6**]. An IOP variation of 1 mmHg produces a change in the central corneal curvature radius of 3 µm. Nocturnal IOP elevation has been associated with body posture changes from sitting to recumbent positions during sleep. Until now, the various methodologies to estimate IOP include single IOP measurement or multiple measurements taken in the clinic during regular office hours to guide treatment [**4**-**6**]. According to various studies, glaucomatous progression occurs despite adequately controlled IOP measured in the office. Hence, to achieve better outcomes in the management of control of IOP, a continuous 24-hour IOP monitoring would be a desirable approach analogous to the 24 h blood pressure and Holter ECG monitoring for arrhythmias, in patients having cardiac illnesses. The 24 hour monitoring of IOP using the common method of IOP measurements such as Goldman Applanation tonometer (GAT), hand held applanation tonometer, pneumotonometer is time consuming and impractical for both patient and ophthalmologist [**7**-**12**]. Additionally, by employing above methods patient’s sleep also gets disturbed with resultant change in patient’s IOP from awakening the patient for measuring IOP even if the patient is admitted in hospital. Therefore, considering all these limitations, it was felt that, at present, there is an unmet need of continuous monitoring of IOP with ways that do not interfere in patients’ activities of daily living.

This review article comprehensively analyzed advent and current status of ways to monitor the 24 hour IOP. A PUBMED search was performed using the 24 Hour tonometry, newer tonometry devices, contact lens sensors, and keywords and all relevant articles were studied. References mentioned in various studied articles were also analyzed. Pertinent articles relating to this article were then appended in references section accompanying this manuscript. 

## Current methods for the 24-hour IOP recording

Currently, there are three methods available, which can provide information about IOP behavior over 24 hours. The three methods are briefly discussed below:

**A. Self tonometry**;

**B. Temporary continuous monitoring device**;

**C. Permanent continuous monitoring device**.

**A. Self tonometry**

Many hand held, portable self-monitoring devices have been proposed and evaluated on basis of ease of use, portability, and safety profile. Tonopen (Reichert) [**13**-**15**] is a portable device that relies on the principle of applanation tonometry. A very good correlation with GAT at physiological pressures was obtained but is not advisable for home self-monitoring, as it requires the use of topical anaesthetic, with antecedent corneal epithelial toxicity. Another instrument, iCare tonometer was designed, which requires the application of external pressure to ocular surface for the creation of visual aura or phosphine. Based on these, Provieweye pressure monitor was introduced in 1998 by Bausch and Lomb, the device having a tip that is applied to superonasal quadrant of lid and patient applies external pressure until a visual aura is obtained and IOP is measured. Although the device is portable, it has a poor correlation with Tonopen and GAT measurement [**16**]. 

Similarly, Rebound tonometry designed by Dekking and Coster (iCare) [**17**,**18**] is also a method consisting of a handheld probe with metallic motion probe and plastic tip in a coil system. The probe accelerates towards cornea with a magnetic field, IOP is measured based on deceleration parameters of the probe as it rebounds from cornea, an average of 6 readings are recorded (maximum and minimum value discarded) before the calculation of an average IOP based on the remaining 4 measurements. No topical anaesthetic is required, and it has strong correlation with GAT but ignores central corneal thickness (CCT), corneal hysteresis, and corneal resistance factors. iCare overestimates IOP in patients with thicker CCT. However, it is more reliable at the peripheral cornea and the measurement independent of corneal curvature [**19**].

Another disadvantage is that it is affected by hand movements and dexterity and cannot take IOP in supine position because probe displacement occurs when device is inverted. To overcome this, a new device called iCare pro was devised, being able to measure IOP in supine position. In a study conducted by Moreno J et al. [**20**], self-monitoring iCare values were compared with GAT in 149 patients with ocular hypertension. The iCare One IOP values were within 3mmHg of corresponding GAT values in 67.1% of the patients, though the differences obtained were not statistically significant. A recent study conducted by Dabasia et al. [**21**] involved a comparison of self obtained, partner obtained and trainer obtained IOP and compared to GAT, after a standardized training, and found that 74% of the patients were able to do self monitoring. It was observed that iCare home underestimates IOP as compared to GAT, with a mean bias of 0.3mmHg (95% CI - 4.6 to 5.2 mmHg) in self obtained group, in partner obtained, bias being 1.1 mmHg (95% CI - 3.2 to 5.5 mmHg) and in training obtained IOP by 1.2 mmHg (95% CI - 3.9 mmHg to 6.3 mmHg). Self-obtained IOP readings were comparable to GAT, although some discrepancies existed between CCT less than 500µ and more than 600µ.

**B. Temporary continuous monitoring devices**

In 1970, use of contact lens to continuously monitor IOP was proposed by Greene and Gilman [**22**]. Various lenses like rigid gas permeable (RGP) results were compared to dynamic contour tonometry in seated position but discomfort was a major disadvantage.

 Sensimed, Switzerland introduced a contact lens sensor (CLS), triggerFISH, to define IOP related changes in the eye. The Sensimed triggerFISH sensor has a disposable silicon contact lens with an embedded micro electrical system, which measures changes in the corneal curvature caused by IOP variation. This is currently approved in Europe and has been recently approved by FDA, USA.

The TriggerFISH has a silicon soft contact lens with a diameter of 14.1 mm and central thickness of 585 µm and has 3 base curves (8.4, 8.7 and 9 mm). Two strain gauges, a microprocessor, and an antenna are embedded within the center. The strain gauges detect changes in corneal shape, with a high correlation between CLS output and imposed IOP. The contact lens receives power from and transmits strain gauge information to an adhesive antenna that is attached to the orbit of a patient. The adhesive antenna sends information to the portable recorder worn by the patients. This TriggerFISH takes about 300 strain gauge readings, over 30 seconds every 5 minutes, over a 24-hour period. The data are sent by bluetooth connection to a computer for analysis. The data points are measured in millivolts or millivolt equivalent relative to the very first reading, which is zero.

**C. Permanent Continuous monitoring device**

IOP company [**23**] (Implandata Ophthalmic Products GmbH Germany) introduced an implantable intraocular device, which is currently undergoing human clinical trials. It is a wireless intraocular transducer (WIT) that has 8 pressure and temperature sensors, an identification and analogue to digital encoder as well as a telemetry unit. Each pressure sensor is composed of 2 parallel plates that indents with change in IOP and sends a signal to the telemetry unit (check). The device can generate a range of settings that allow the monitoring at variable intervals. In vitro studies with the device have revealed that the Implandata EyemateR is biocompatible with a good subjective tolerance in rabbit eyes for up to 25 months. This was confirmed by the lack of intraocular toxicity on histopathology and that when device was immersed in saline solution for 4 years it remained functional showing its efficacy in aqueous medium.

In ARGOS study [**24**], patients with well controlled POAG/ NTG with visually significant cataract were operated for the cataract with Eyemate placement in the sulcus, confirmed by ultrasonic biomicroscopy. It was found that even at 1-year postoperative period, all patients had controlled glaucoma and none of them had pupillary block, macular edema, and visual deterioration. Even CCT and endothelial cell count were within normal limits but telemetric readings were performed as compared to constant GAT, which is speculated to be due to antenna problems.

A brief review of TriggerFISH Contact Lens System (CLS) is shown below.

**TriggerFISH CLS - overview**

The major advantage of CLS is that the patient IOP measurements can be done while the patient is ambulatory minimally affecting their daily routine. The information received can be used for providing appropriate feedback especially concerning their lifestyle, blinks, and effect of treatment. The major current disadvantage is that the readings are in milli volt equivalent rather than mmHg; it is very difficult to convert mVolt to mmHg, as equivalent pressure and volume are non-linear and is influenced by viscoelastic properties of the eye. Hence, new algorithms are required for conversion. Currently, additional limitations include high cost of the device and issues pertaining to patient acceptance of this device.

**Tolerability**

In a prospective study conducted by Lorenz et al. [**25**], in 20 glaucoma patients and 20 aged matched healthy controls, tolerability of CLS was assessed using a Visual Analog Scale (VAS), where zero indicated no discomfort and 100 as severe discomfort. VAS was 21.82 in healthy controls and 26.8 in glaucoma cohort (p=0.44). One healthy subject was removed because of improper device fitting secondary to steep corneal radii and one glaucoma patient developed discomfort due to immediate pain and foreign body sensation (due to improper encapsulation of microelectronic components in the sensor). More than 95% of the subjects showed willingness to use the device, which was encouraging. In another study conducted by Mansouri et al. [**26**], 21 glaucoma suspects and 19 glaucoma patients participated in the 24-hour IOP monitoring sessions timed 1 week apart. Tolerability during first session and second session using VAS was 27.2 and 23.8 respectively (p=0.22). No statistically significant correlation was found between VAS score and glaucoma status (r=0.14; p=0.23). 4 patients reported poor tolerability during first monitoring (VAS > 54) and 3 patients reported poor tolerability during second monitoring session.

In another study conducted by Agullo et al. [**27**] to determine the difference in relative IOP measured by Sensimed TriggerFISH CLS in flat as compared to 300 head up sleeping positions in patients with progressive POAG or normotensive glaucoma based on recurrent disc haemorrhage. There was an increase of 6 mmHg of IOP from sitting to supine position in healthy subjects and subjects with glaucoma. This effect may play a role in the progression of glaucoma. This elevation of IOP has been correlated with changes in episcleral venous pressure and ophthalmic arterial pressure.

One important disadvantage of TriggerFISH is that corneal swelling occurs during sleep and can be aggravated by overnight use of contact lens. Increase in CCT, presumably due to corneal swelling with overnight CLS wear was not significant to explain increased signal output. However, changes in corneal curvature and corneal irregularities may induce alteration in corneoscleral junction angulation, thereby modifying signal output in healthy subjects who were exposed to 24 hours CLS wear four times. Another disadvantage is that it gives values of IOP in mVEq and there is no reliable conversion chart available to corresponding mm Hg values.

Agullo et al. [**27**] also compared IOP spikes with triggerFISH during REM sleep and NREM sleep and observed increased IOP spikes during REM presumably due to increased ocular movements as compared to NREM sleep, which is associated with decreased ocular movements and increased parasympathetic activity thereby leading to decreased blood pressure and heart rate and associate decreased IOP. There is also variability in IOP measured during CLS wear and 24 hour after removal of CLS in one eye and IOP measurement with other methods in other eye. In 2015, Mansouri et al. [**28**] measured the agreement between CLS output in 1 eye and pneumotonometry taken at every 2 hours in the fellow eye in 33 subjects, both of these yielding a positive Pearson value of 0.956 thereby showing a high correlation.

**Circadian pattern of IOP using CLS**

Tojo et al. [**29**] conducted a study of monitoring the 24 hour CLS in patients with pseudoexfoliation and 11 healthy subjects, finding a greater range of IOP in pseudoexfoliation compared to normal. All healthy subjects had nocturnal increase in IOP but only 7 pseudoexfoliation had nocturnal IOP increase. Tan et al. [**30**] evaluated 25 patients with PACG for 24 hours using CLS, and found that nocturnal peak in CLS was obtained in PACG along with a decrease in the morning and a further decrease during daytime. Agnifilli et al. [**31**] studied the 24 hour CLS in 10 POAG, 10 NTG and 10 healthy subjects, all having a nocturnal increased IOP but a maximum amplitude of sensor occurred in POAG patients. Tojo et al. [**32**] measured the 24 hours CLS in 12 healthy subjects, and 10 NTG patients. A significant increase in IOP in NTG patients was noticed during both diurnal and nocturnal period vs. healthy subjects.

**CLS use in other morbidities**

Parekh et al. [**33**] studied the 24 hours CLS in 10 patients with TED and found that all had significant nocturnal IOP peak and 20% also had a IOP spike when awake around 6:30 am. Overall, no significant increase in CLS signal output was observed from wake to sleep transit during their study cohort. 

**CLS use and studying effect of treatment**

Surgical efficiency can also be tested using CLS as was done by Rekas et al. [**34**], 10 patients underwent IOP monitoring using CLS pre and post canaloplasty at 3 and 12 months postoperatively. A significant change in IOP amplitude was observed between pre and 3 month post canaloplasty (p=0.027) and between pre and 12 months post canaloplasty (p=0.031).

Lee et al. [**35**] conducted a 24 hours CLS in 18 patients, one week prior and 1 month following SLT (Selective Laser Trabeculoplasty). Participants were in 2 groups, a SLT success group with participants achieving ≥ 20% reduction in GAT measurements one month after SLT and a post SLT non-success group. It was observed that IOP-related pattern amplitude was reduced in NTG patients after undergoing successful SLT treatment, whereas the non-success group exhibited an increase of pattern amplitude. Higher 24-hour CLS pattern variability was observed in non-success patients 1 month post-SLT.

## Conclusion

With the advent of new technologies and growing emphasis on bioinformatics, customized treatment regimens will become the standard of care of glaucoma. A reliable, accurate, 24-hour IOP monitoring device will provide a novel understanding of patients IOP behavior, and circadian pattern in IOP. There are new challenges to be circumvented in the form of novel methods of data collection, conversion of mVolt data into mm Hg, tolerance with long-term use, patient acceptability, ability of the device to fit in eye having anterior filtering blebs, and cost per device to the patient. Currently, there is also no consensus whether IOP lowering medicines can or cannot be used with the CLS on. In time, these challenges will be gradually addressed and the patients would be offered a variety of options for the 24-hour IOP monitoring.

According to current literature, TriggerFISH CLS is a safe device for IOP in healthy and glaucoma subjects. It helps providing knowledge about control of IOP by providing relevant information about the 24-hour IOP pattern. The utility of this device in relation to progressive vision loss is a matter of future study.

**Acknowledgement**

The authors would like to thank Sonam Juneja, Gursimran Chahal, Sukarma Singh, and Soumya Sharma for their logistic and technical help in the preparation of this manuscript.

**Sources of Funding**

None.

**Financial disclosure**

None of authors of this article have any financial interest in the devices alluded to in this article.

**Conflicts of Interest**

Nil.
